# Synthesis and Characterization of Some New 4-Hydroxy-coumarin Derivatives

**DOI:** 10.3390/molecules190811791

**Published:** 2014-08-07

**Authors:** Yasameen K. Al-Majedy, Abdul Amir H. Kadhum, Ahmed A. Al-Amiery, Abu Bakar Mohamad

**Affiliations:** 1 Department of Chemical and Process Engineering, University Kebangsaan Malaysia (UKM), Bangi, Selangor 43000, Malaysia; E-Mails: amir@eng.ukm.my (A.A.H.K.); dr.ahmed1975@gmail.com (A.A.A.-A.); drab@eng.ukm.my (A.B.M.); 2 Environmental Research Center, University of Technology (UOT), Baghdad 10001, Iraq

**Keywords:** bromoacetate, 4-hydroxycoumarin, maleic anhydride, mercaptoacetic acid

## Abstract

Some novel coumarins were synthesized starting from 4-hydroxycoumarin and methyl bromoacetate. The structures of the newly obtained compounds were confirmed by elemental analysis, mass, IR and NMR spectra.

## 1. Introduction

Coumarins or benzo-2-pyrone derivatives are one of the most significant families of natural product compounds and are also important in synthetic organic chemistry. They have been widely used as starting materials or intermediates in the pharmaceutical, perfumery and agrochemical industries. Coumarins are also used as fluorescent brighteners, efficient laser dyes and additives in food and cosmetics [1]. The coumarins represent a large group of compounds that have been reported to possess a wide range of biological activities [2,3,4], including anticoagulant and antithrombotic properties [5,6,7]. Many coumarin derivatives, especially 4-hydroxycoumarin, show significant anticoagulant action by antagonizing the action of vitamin K. [8,9]. Recently, coumarins have attracted considerable attention for electronic and photonic applications [10,11] due to their inherent photochemical characteristics, reasonable stability and solubility in various organic solvents. Many coumarin derivatives have been commercialized as blue-green lasers for fluorescent labels, fluorescent probes [12,13,14] and enzymatic measurements [15]. They exhibit intense fluorescence upon substitution with various functional groups at different positions [16,17].

**Scheme 1:**
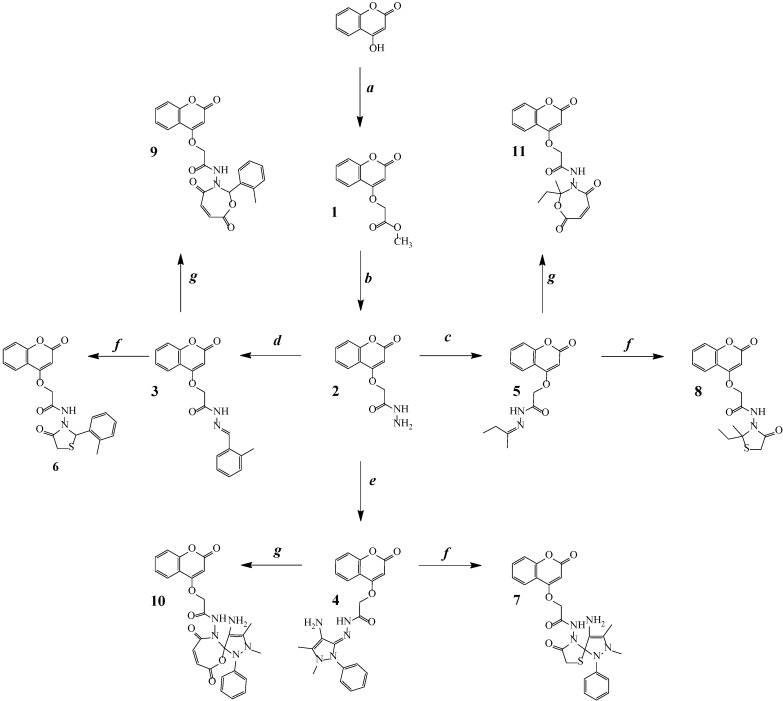
Reaction sequences of the synthesized compounds.

In view of the high degree of bioactivity shown by 4-aminoantipyrine, thiazolidinones and hydroxycoumarin heterocyclic analogs, and in continuation of previous studies [18,19,20,21,22,23], we focus herein on the design of some novel structural entities that incorporate both of these moieties in a single molecular scaffold. We first report the synthesis of methyl 2-(coumarin-4-yloxy)acetate (**1**), which was then used as starting material for the synthesis of new molecules **2**–**11** (Scheme 1).

*Reagents and Conditions*: *a* = Ethyl bromoacetate/reflux; b = hydrazine hydrate/refluxed; Ethylmethylketone/refluxed in ethanol; d = 2-methyl benzaldehyde/refluxed in ethanol; e = 4-aminoantipyrine/refluxed in ethanol; f = mercaptoacetic acid/refluxed in benzene; g = maleic anhydride/refluxed in benzene.

## 2. Results and Discussion

### 2.1. Chemistry

The reaction sequences for the synthesis of coumarins **2**–**11** starting from 4-hydroxycoumarin are outlined in Scheme 1. Methyl 2-(coumarin-4-yloxy)acetate (**1**) was obtained by refluxing methyl bromoacetate with 4-hydroxycoumarin in anhydrous acetone in the presence of anhydrous potassium carbonate. The FT-IR spectrum of this compound showed an absorption band at 1,723.1 cm^−1^ (ester C=O carbonyl stretching). The ^1^H-NMR spectrum exhibited a singlet at δ 3.63 ppm due to the three CH_3_ protons. The reaction of **1** with hydrazine hydrate afforded hydrazide **2** in good yield. The FT-IR spectrum of compound **2** showed absorption bands at 3,233.3 and 3,210.0 cm^−1^ (hydrazide NH-NH_2_). The ^1^H-NMR spectrum exhibited a singlet at δ 4.45 ppm due to the two CH_2_ protons and a singlet due to the single NH proton at δ 8.21 ppm. The reaction of compound **2** with various carbonyl compounds (2-methylbenzaldehyde, 4-aminoantipyrine and ethyl methyl ketone) yielded the new Schiff bases *N'*-(2-methylbenzylidene)-2-[(coumarin-4-yl)oxy]acetohydrazide (**3**), *N'*-[4-amino-1,5-dimethyl-2-phenyl-1*H*-pyrazol-3(2*H*)-ylidene]-2-[(coumarin-4-yl)oxy]acetohydrazide (**4**) and *N'*-(butan-2-ylidene)-2-[(coumarin-4-yl)oxy]acetohydrazide (**5**), respectively. Schiff bases **3**, **4** and **5** were cyclized by reaction with mercaptoacetic acid to yield novel coumarin compounds *N*-[4-oxo-2-(O-tolyl)thiazolidin-3-yl]-2-[(coumarin-4-yl)oxy]acetamide (**6**), *N*-(4-amino-2,3-dimethyl-8-oxo-1-phenyl-6-thia-1,2,9-triazaspiro[4.4]non-3-en-9-yl)-2-[(coumarin-4-yl)oxy]acetamide (**7**) and *N*-(2-ethyl-2-methyl-4-oxothiazolidin-3-yl)-2-[(coumarin-4-yl)oxy]acetamide (**8**), respectively. The FT-IR spectrum of compound **6** showed absorption bands at 3,191.4 cm^−1^ (NH) and 1,715.6 cm^−1^ (C=O, lactone) as well as 1,695 and 1,677 cm^−1^ (C=O, amide). The ^1^H-NMR spectrum exhibited a singlet due to the (S-CH) proton at δ 4.88 ppm and another singlet due to the O-CH_2_ proton at δ 5.11 ppm (2H). For compound **7**, the FT-IR spectrum showed absorption bands at 3,388.3 cm^−1^ for NH_2_ and 3,189.1 cm^−1^ for N-H, and the C=N bands disappeared due to cyclization. The ^1^H-NMR spectrum exhibited a doublet due to the two protons at δ 4.89 and δ 4.12 ppm and singlet due to the single S-CH proton at δ 3.89 ppm. For compound **8**, the FT-IR spectrum showed absorption bands at 3,199.0 cm^−1^ (NH) as well as 1,691 and 1,685 cm^−1^ (C=O, amide); the C=N bands disappeared due to cyclization. The ^1^H-NMR spectrum exhibited a doublet due to the S-CH_2_ proton at δ 3.86 and δ 8.02 ppm, and a singlet due to the NH proton. Another three novel compounds were synthesized by the cyclization of Schiff bases **3**, **4** and **5** with maleic anhydride to yield *N*-[4,7-dioxo-2-(O-tolyl)-1,3-oxazepin-3(2*H*,4*H*,7*H*)-yl]-2-[(coumarin-4-yl)oxy] acetamide (**9**), *N*-[2-(4-amino-1,5-dimethyl-2-phenyl-2,3-dihydro-1*H*-pyrazol-3-yl)4,7-dioxo-1,3-oxazepin-3(2*H*,4*H*,7*H*)-yl]-2-[(coumarin-4-yl)oxy] acetamide (**10**) and *N*-[2-ethylmethyl-4,7-dioxo-1,3-oxazepin-3(2*H*,4*H*,7*H*)-yl]-2-[(coumarin-4-yl)oxy] acetamide (**11**), respectively. For compound **9**, the FT-IR spectrum showed absorption bands at 3,196.2 cm^−1^ (NH) and 1,667.9 cm^−1^ for the amide as well as 1,755 and 1,745 cm^−1^ for carbonyls. The ^1^H-NMR spectrum exhibited a singlet at δ 5.38 ppm due to the two O-CH_2_ protons. For compound **10**, the FT-IR spectrum of showed absorption bands at 2,981.4 cm^−1^ (C-H, aliphatic), 1,765 and 1,734 cm^−1^ for the carbonyls, and 1,678 cm^−1^ for the amide. The ^1^H-NMR spectrum exhibited a singlet δ 4.89 ppm due to the two O-CH_2_ protons at. For compound **11**, the FT-IR spectrum of showed absorption bands at 3,199 cm^−1^ (NH) and 1,669.2 cm^−1^ for the amides as well as 1,758 and 1,733 cm^−1^ for the carbonyls. The ^1^H-NMR spectrum exhibited a singlet due to the two O-CH_2_ protons at δ 4.65 ppm.

### 2.2. Geometrical Isomers of the Acylhydrazone of Coumarins ***3**–**5***

**Figure 1:**
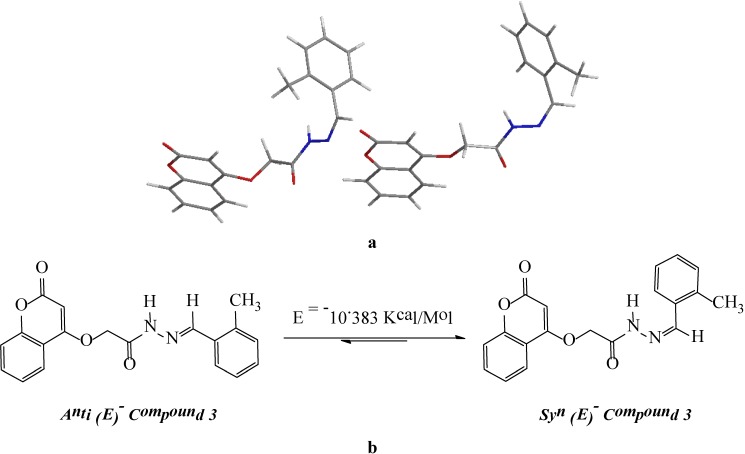
Probable conformational isomers of the *N*-acylhydrazone of compound **3**. (**a**) = 3dimetional structures; (**b**) = *geometrical Isomers)*.

With respect to the C=N double bond, *N*-acylhydrazones (NAHs) may exist as *Z*/*E* geometrical isomers and *syn*/*anti* amide conformers [24,25]. In a study involving compounds **3**, **4** and **5**, energy calculations performed on the selected conformers according to the density functional theory (DFT) B3LYP method using the 6–31G basis set by means of the Gaussian 09, revision A.02 method indicated a slight difference in energy (ΔE = −10.383 Kcal/mol, −2.350 Kcal/mol and −13.918 Kcal/mol) respectively, between the *syn*-periplanar and *anti*-periplanar conformers in the favor of the former (Figure 1). Therefore, we concluded that the new derivatives **3**, **4** and **5** were obtained as single *E* geometrical isomers.

## 3. Experimental Section

### 3.1. General Information

The chemicals used during synthesis were supplied by Sigma-Aldrich (Selangor, Malaysia). The IR spectra were obtained on a Nicolet 6700 FT-IR spectrophotometer (Thermo Nicolet Corp., Madison, WI, USA), and the values are expressed in cm^−1^. Nuclear magnetic resonance (NMR) spectra were recorded using an AVANCE III 600 MHz spectrometer (Bruker, Billerica, MA, USA), using DMSO as an internal standard and the values are expressed in δ ppm. Elemental microanalysis was performed on an Elementar Vario El III Carlo Erba 1108 elemental analyzer (Carlo Erba Reagenti SpA, Rodano, Italy).

### 3.2. Methyl 2-(coumarin-4-yloxy)acetate *(**1**)*

A suspension of 4-hydroxycoumarin (0.999 g, 6.17 mmol) in acetone (30 mL) was refluxed with methyl bromoacetate (9.15 mmol) and K_2_CO_3_ (4.69 g, 33.91 mmol) for 12 h. After cooling, the mixture was evaporated to dryness and the residue was partitioned between CHCl_3_ (50 mL) and water (50 mL). The organic phase was dried using Na_2_SO_4_, filtered and evaporated to dryness. The residue was recrystallized from acetone; yield 85%; m.p. 84–85 °C; ^1^H-NMR (CDCl_3_): δ 3.6 (s, 3H, CH_3_), 4.79 (s, 2H, CH_2_) and 5.58 (s, 1H, -C=C-H), 7.3111, 7.555, 7.896 (three s, 1H each, aromatic ring); IR (cm^−1^): 2,960 (C-H, aliphatic), 3,083.4 (C-H, aromatic), 1,760.3 (C=O, ester), 1,723.1 (C=O, lactone), 1,624.5 (C=C, alkene), 1,567.2 (C=C, aromatic).

### 3.3. 2-(Coumarin-4-yloxy)acetohydrazide *(**2**)*

A solution of compound **2** (2.34 g, 10 mmol) in ethanol 25 mL was refluxed with hydrazine hydrate (15 mmol) for 4 h. After concentrating the reaction mixture an oily mass separated out and was recrystallized using ethanol, yield 55%; ^1^H-NMR (CDCl_3_): δ 4.45 (s, 2H, CH_2_), 4.75 (s, 2H, NH_2_), 5.43 (s, 1H, -C=C-H), 7.41–7.78 (m, 4H, aromatic ring), 8.21 (s, 1H, NH); IR (cm^−1^): 3,233.3, 3,210 (N-H), 2,959.0 (C-H, aliphatic), 3,083.9 (C-H, aromatic), 1,721.4 (C=O, lactone), 1,624.2 (C=O, amide).

### 3.4. Synthesis of Schiff bases *3–5*

A solution of compound **2** (0.2 mmol) in ethanol (25 mL) was refluxed with 2-methyl benzaldehyde, 4-aminoantipyrine or ethyl methyl ketone (0.2 mmol) for 20 h. After cooling to room temperature, a solid mass was filtered and the solid was recrystallized from ethanol.

*N′-(2-Methylbenzylidene)-2-(coumarin-4-yloxy)acetohydrazide* (**3**). Yield 50%; ^1^H-NMR (CDCl_3_): δ 2.891 (s, 3H, CH_3_), 4.19 (s, 2H, CH_2_), 5.355 (s, 2H, O-CH2), 5.57 (s, 1H, -C=C-H), 7.270–7.75 (m, 4H, aromatic ring); 8.31 (s, 1H, N=CH), 8.01 (s, 1H, NH); IR (cm^−1^): 3,199.3 (N-H), 2,965.4 (C-H, aliphatic), 3,067.2 (CH, aromatic), 1,730.3 (C=O, lactone), 1,683.7 (C=O, amide), 1,627.7 (C=C).

*N'-(4-Amino-1,5-dimethyl-2-phenyl-1H-pyrazol-3(2H)-ylidene)-2-(coumarin-4-yloxy)acetohydrazide* (**4**). Yield 60%; m.p 162-163 °C; ^1^H-NMR (CDCl_3_): 4.05 (s, 2H, assignment?), 4.67 (s, 2H, O-CH_2_), 5.23 (s, 1H, -C=C-H), 3.21 (s, 3H, CH_3_), 2.83 (s, 3H, CH_3_)_, _ 7.11–7.34 (m, 4H, aromatic ring), 8.0 (s, 1H, NH), 8.41 (s, 1H, N=CH); IR (cm^−1^): 3,432.8 (NH_2_), 3,328.0.3 (N-H), 2,989.9 (C-H, aliphatic), 3,077.3 (C-H, aromatic), 1,725.8 (C=O, lactone), 1,651.9 (C=O, amide), 1,616.3 (C=N), 1,6229 (C=C); Analysis: Calc. for C_21_H_21_N_5_O_4_: C 61.91%, H 5.20%, N 17.19%. Found: C 60.057%, H 5.641%, N 16.685%.

*N′-(Butan-2-ylidene)-2-(coumarin-4-yloxy)acetohydrazide* (**5**). Yield 50%; m.p 71–72 °C; ^1^H-NMR (CDCl_3_): δ 0.81 (t, 3H, CH_3_), 1.49 (q, 2H, CH_2_), 2.01 (s, 3H, CH_3_), 4.64 (s, 2H, O-CH_2_), 5.21 (s, 1H, -C=C-H), 6.95 (s, 1H, N=CH), 7.38–7.79 (m, 4H, aromatic ring); IR (cm^−1^): 3,198.2 (N-H), 2,922.9 (C-H, aliphatic), 3,068.4 (C-H, aromatic), 1,732.2 (C=O, lactone), 1,673.1 (C=O, amide), 1,615.3 (C=N), 1,621.5 (C=C); Analysis: Calc. for C_15_H_16_N_2_O_4_: C 62.49%, H 5.59%, N 9.72%. Found: C 61.70%, H 4.89%, N 9.11%.

### 3.5. Cyclization with Mercaptoacetic Acid: Synthesis of Compounds ***6**–**8***

A mixture of compound (**3** or **4** or **5**) (0.01 mole) with mercaptoacetic acid (0.01 mole) in dry benzene (50 mL) was refluxed in a water bath for 20 h, filtered off, washed with water, dried and recrystallized from dichloromethane.

*N-[4-Oxo-2-(o-tolyl)thiazolidin-3-yl)-2-(coumarin-4-yloxy]acetamide* (**6**). Yield 45%; oil; ^1^H-NMR (CDCl_3_): δ 2.920 (s, 3H, CH_3_), 4.88 (s, S-CH), 4.67 (s, 1H, CH), 5.11 (s, 2H, O-CH_2_), 5.73 (s, 1H, -C=C-H), 7.270–7.820 (m, 4H, aromatic ring), 8.12 (s, 1H, NH); IR (cm^−1^): 3,191.4 (N-H), 2,930 (C-H, aliphatic), 3,043 (C-H, aromatic), 1,715.6 (C=O, lactone), 1,695, 1677 (C=O, amide), 1,631.5 (C=C, aromatic).

*N-{2-[(S)-4-Amino-1,5-dimethyl-2-phenyl-2,3-dihydro-1H-pyrazol-3-yl]-4-oxothiazolidin-3-yl}-2-(coumarin-4-yloxy)acetamide* (**7**). Yield 45%; oil; ^1^H-NMR (CDCl_3_): δ 2.24 (s, 3H, CH_3_), 3.06 (s, 3H, N-CH_3_), 3.89 (s, S-CH_2_), 4.67 (s, 2H, CH_2_), 4.69 (s, 2H, O-CH_2_), 5.26 (s, 1H, -C=C-H), 7.31, 7.52, 7.81 (s, 1H, aromatic ring), 6.84, 7.11, 7.29 (s, 1H, aromatic ring) 8.02 (s, 1H, NH), 8.32 (s, NH_2_); IR (cm^−1^): 3,388.3 (NH_2_), 3,189.1 (N-H), 2,928 (C-H, aliphatic), 3,057.7 (C-H, aromatic), 1,719.5 (C=O, lactone), 1,685, 1667 (C=O, amide), 1,633.7 (C=C, aromatic); Analysis: Calc. for C_25_H_25_N_5_O_5_S: C 59.16%, H 4.96%, N 13.80%. Found: C 58.91%, H 4.11%, N 12.92%.

*N-(2-Ethyl-2-methyl-4-oxothiazolidin-3-yl)-2-(coumarin-4-yloxy)acetamide* (**8**). Yield 45%; oil; ^1^H-NMR (CDCl_3_): δ 0.89 (t, 3H, CH_3_), 1.59 (s, 3H, CH_3_), 1.78 (q, 2H, CH_2_), 3.86 (s, S-CH), 4.68 (s, 2H, O-CH_2_), 5.21 (s, 1H, -C=C-H), 7.36, 7.54, 7.71 (s, 1H, aromatic ring) 8.02 (s, 1H, NH); IR (cm^−1^): 3,199.0 (N-H), 2,931.3 (C-H, aliphatic), 3,040.5 (C-H, aromatic), 1,719 (C=O, lactone), 1,691, 1,685 (C=O, amide), 1,632 (C=C, aromatic).

### 3.6. Cyclization with Maleic Anhydride; Synthesis of Compounds ***9**–**11***

A mixture of compound (**3**, **4** or **5**) (1 mmol) and maleic anhydride (1 mmol) in dry benzene (50 mL) was refluxed in a water bath for 20 h. The solvent was removed and the precipitate was recrystallized from tetrahydrofuran.

*N-[4,7-Dioxo-2-(o-tolyl)-1,3-oxazepin-3(2H,4H,7H)-yl]-2-(coumarin-4-yloxy) acetamide* (**9**). Yield 40%; m.p. 128–130 °C; ^1^H-NMR (CDCl_3_): δ 2.784 (s, 3H, CH_3_), 3.012 (d, 1H, CH), 3.69 and 4.02 (dd, 1H, CH=CH), 5.38 (s, 2H, O-CH_2_), 6.281 (s, 1H, -C=C-H), 7.329, 7.446, 7.964 (s, 1H, aromatic ring) 9.14 (s, 1H, NH); IR (cm^−1^): 3,196.2 (N-H), 2,970.5 (C-H, aliphatic), 3,087.2 (C-H, aromatic), 1,755, 1,745 (C=O), 1,667.9 (C=O, amide), 1,630.1 (C=C, aromatic).

*N-[2-(4-Amino-1,5-dimethyl-2-phenyl-2,3-dihydro-1H-pyrazol-3-yl)4,7-dioxo-1,3-oxazepin-3(2H,4H,7H)-yl]-2-(coumarin-4-yloxy) acetamide* (**10**). Yield 35%; m.p 120–121 °C; ^1^H-NMR (CDCl_3_): δ 2.24 (s, 3H, CH_3_), 3.01 (s, 3H, CH_3_), 4.89 (s, 2H, O-CH_2_), 5.38 (s, 1H, -C=C-H), 6.89 and 6.39 (dd, 1H, CH=CH), 6.99, 7.11, 7.29 (s, 1H, aromatic ring); 8.14 (s, 1H, NH), 8.45 (s, NH_2_); IR (cm^−1^): 3,391.8 (NH_2_), 3,284.1 (N-H), 2,981.4 (C-H, aliphatic), 3,081.2.2 (C-H, aromatic), 1,765, 1,734 (C=O), 1,678 (C=O, amide), 1,629.6 (C=C, aromatic); Analysis: Calc. for C_27_H_25_N_5_O_7_: C 61.01%, H 4.74%, N 13.18%. Found: C 60.73%, H 4.01%, N 12.65%.

*Synthesis of N-[2-Ethylmethyl-4,7-dioxo-1,3-oxazepin-3(2H,4H,7H)-yl]-2-(coumarin-4-yloxy) acetamide* (**11**). Yield 40%; m.p. 118–120 °C; ^1^H-NMR (CDCl_3_): δ 0.89 (t, 3H,CH_3_), 1.69 (s, 3H, CH_3_), 1.505 (q, 2H, CH_2_), 4.65 (s, 2H, O-CH_2_), 5.23 (s, 1H, -C=C-H), 6.82 and 6.41 (dd, 1H, HC=CH), 7.41, 7.453, 7.81 (s, 1H, aromatic ring), 8.12 (s, 1H, NH); IR (cm^−1^): 3,199.0 (N-H), 2,973.1 (C-H, aliphatic), 3,091.0 (C-H, aromatic), 1,758, 1,733 (C=O), 1,669.2 (C=O, amide), 1,630.3 (C=C, aromatic); Analysis: Calc. for C_19_H_18_N_2_O_7_: C 59.07%, H 4.70%, N 7.25%. Found: C 58.81%, H 4.12%, N 6.88%.

## 4. Conclusions

A series of new cyclic compounds based 4-hydroxycoumarin were successfully synthesized in high to acceptable yields (35%–85%). The proposed structures of the new coumarins were confirmed by spectral analysis performed by IR, UV-Vis, ^1^H-NMR and elemental analysis. Coumarins **6**, **7** and **8** were synthesized by the cyclization of Schiff bases **3**, **4** and **5** using mercaptoacetic acid. The cyclization of Schiff bases **3**–**5** with maleic anhydride yielded new coumarins **9**–**11**.
